# Differential role of beta band activity in a dual-task working memory paradigm under internally vs. externally directed cognition

**DOI:** 10.3389/fnhum.2026.1791453

**Published:** 2026-05-12

**Authors:** Ankit Yadav, Arpan Banerjee, Dipanjan Roy

**Affiliations:** 1 National Brain Research Centre, Gurugram, India; 2 Indian Institute of Technology, Jodhpur, India

**Keywords:** beta band activity, dual-task paradigm, internally directed cognition, quantile regression, working memory

## Abstract

Internally directed cognition (IDC), whether spontaneous or intentional, has been associated with impaired performance in cognitive tasks. Yet, the neurophysiological mechanisms underpinning this disruption remain poorly understood. In the present study, we characterized the neural correlates of IDC and identified how they impact on performance in a color-recall working memory task using electroencephalography (EEG). Participants performed a novel dual-task paradigm involving either self-referential (IDC) or perceptual processing of adjectives, involving externally directed cognition (EDC) followed by a color-recall task. IDC enhanced late frontal positivity in EEG over medial-frontal electrodes as a marker of sustained self-referential processing. Time–frequency analyses further revealed increased event-related desynchronization in alpha and beta bands during stimulus encoding in the IDC condition, as well as increased alpha synchronization during the delay period, consistent with internal attention maintenance. To capture trial-level variability in task performance, we applied conditional quantile regression to individual trial-level observations. Results showed that beta desynchronization in interaction with condition type during encoding influenced performance significantly in trials with low errors, whereas trials with high error in color recall were better explained by increased reaction times. These findings provide converging electrophysiological evidence for distinct neural signatures of internally directed cognition and highlight their behavioral consequences in working memory performance.

## 1 Introduction

Attention continuously shifts between the external world, for example, counting something on the screen, and the internal world, reflecting on feelings about own mental state shaped by last night’s dinner. Complex cognitive functions rely on such seamless interplay between external attention and internally generated thought processes. According to Dixon et al. (2014), internally directed cognition (IDC) involves internally directed attention towards information in long-term memory, working memory, prospective and retrospective thinking, and self-referential content. This includes episodic memory retrieval, simulating future events, stimulus-independent thoughts, mental imagery, and dreaming. IDC is largely decoupled from the external environment, operating independently or in response to internal or external stimuli (see Chun et al., 2011 for review). In contrast, the externally directed cognition (EDC) refers to the attention directed towards the external perceptual world and involves processing sensory information in a specific modality, such as visual and auditory input (Chun et al., 2011; Dixon et al., 2014). While internally focused states are essential for introspection and self-evaluation, when we have an EDC task at hand, switching to such states during encoding and maintenance phases of the task may detract the cognitive resources necessary for processing and encoding external stimuli, ultimately impairing performance on the task that require sustained attention (Kam and Handy, 2014; Kane and McVay, 2012; Killingsworth and Gilbert, 2010; Rummel and Boywitt, 2014; Smallwood et al., 2008).

To probe how these limited cognitive resources are allocated, working memory paradigms frequently employ dual-tasking. Dual-tasking creates structural bottlenecks and overlapping neural demands, leading to behavioral costs (Kiesel et al., 2010; Pashler, 1994; Ruthruff et al., 2001; Worringer et al., 2019). Competing tasks, whether motor, spatial, or auditory, have been shown to severely disrupt simultaneous visual processing and spatial maintenance (Romeo et al., 2019). Furthermore, recent high-density electroencephalography (EEG) study highlights that concurrent distractions specifically impair the encoding phase of visual memory (Del Popolo Cristaldi et al., 2025). While the impact of external distractions on working memory is well-documented (Clapp et al., 2010; Clapp and Gazzaley, 2012; Gresch et al., 2021; Hallenbeck et al., 2021; Zickerick et al., 2020), the specific dual-task cost imposed by an internal distractor remains poorly understood.

Self-referential processing, a central aspect of IDC, plays a crucial dual role in cognition by shaping subjective experience and modulating task performance by promoting internally generated thoughts—often at the expense of task-focused attention (Dixon et al., 2014; Huijser et al., 2018). Neuroimaging studies show self-referential processing not only recruits the default mode network (DMN) and cortical midline structures but also interacts dynamically with attention and control networks (Benedek et al., 2016; Dixon et al., 2014). The balance between EDC and IDC is mediated by the interplay among the DMN, which supports internally generated thought, and frontoparietal control and dorsal attention networks, which support goal-directed and perceptual processing. When self-relevance is high, self-referential processing can shift this balance toward internally focused cognition (Raichle et al., 2001; Zhao et al., 2018). Beyond neuroimaging evidence, event-related potential (ERP) studies have consistently documented enhanced late positivities for stimuli that are motivationally significant or self-relevant (Hajcak and Foti, 2020; Katyal et al., 2020). While passive exposure to emotionally arousing or self-relevant stimuli typically elicits a canonical, posteriorly distributed late positive potential associated with heightened visual attention (De Cesarei and Codispoti, 2011), the scalp distribution of these late components varies significantly depending on specific cognitive demands. Notably, a distinct late frontal positivity is frequently observed when paradigms explicitly require participants to actively evaluate self-relevance or retrieve self-related information from memory (Fields and Kuperberg, 2012; Kotlewska and Nowicka, 2016; Magno and Allan, 2007; Mao et al., 2017; Nowicka et al., 2018; Porter et al., 2021). Because internally directed cognition intrinsically demands the active evaluation, retrieval, and maintenance of internal thoughts, this late frontal positivity may serve as a critical temporal marker for the allocation of cognitive resources toward internal representations rather than external sensory processing.

In oscillatory dynamics, particularly in alpha and theta band, differential processing has been reported in IDC and EDC (Kam et al., 2018; Kam and Handy, 2014; Mu and Han, 2010; Subramaniam et al., 2019). Posterior alpha band activity is reported to index both internal and external modes of cognition. EDC involving shifts of visuospatial attention reliably modulate posterior alpha power as it is reported to decreases over cortical regions processing attended inputs and increases over unattended regions (Rihs et al., 2009; Sauseng et al., 2005; Thut et al., 2006; Worden et al., 2000). Internally directed cognitive states, such as self-referential thought (Mu and Han, 2010), IDC in steady state visual evoked potentials (SSVEP) task (O’Connell et al., 2009), and mind wandering (Kam et al., 2018) tend to increase alpha band activity over parietal sites. Frontocentral midline theta power is often associated with successful performance in various externally directed tasks (Cavanagh and Frank, 2014; Kam et al., 2018, 2022). Research on attentional reorientation suggests that beta power increase plays a role in redirecting attention from internal distractions (e.g., stress-induced intrusive thoughts) to external tasks (Palacios-García et al., 2021), while beta desynchronization is associated retrieval of self-generation information (Graber and Fujioka, 2020; Subramaniam et al., 2019).

Despite these advances, some aspects of the impact of IDC on complex cognitive tasks remain unexplored. Specifically, it is unclear whether IDC affects the encoding of perceptual features when cognitive resources are limited, the rehearsal and recall during the maintenance period, or both processes simultaneously. Moreover, although prior research has identified EEG signatures associated with IDC, it is still not known which of these neural features specifically drive its detrimental impact on task performance. To address this gap, the present study investigated the resource competition between IDC and EDC using a dual-task paradigm involving a self-referential IDC or a perceptual EDC task paired with a color-recall working memory task. Considering this, the study employed a novel color-recall working memory paradigm in which participants viewed personality adjectives and either judged how well each word described them (IDC condition) or counted the number of vowels in the word (EDC condition), while EEG activity was recorded. The goal of the analysis was to isolate the temporal and spectral EEG signatures of IDC and evaluate their predictive impact on trial-by-trial working memory performance. Specifically, we formulated our investigation around three primary objectives. First, regarding the behavioral dual-task cost, we hypothesized that the IDC condition would induce a greater color-recall error compared to the EDC condition, driven by the resource-intensive nature of self-referential processing (Huijser et al., 2018). Second, concerning the neural signatures of these cognitive states, we predicted that during stimulus encoding, the IDC condition would elicit enhanced late frontal positivity, reflecting self-referential engagement (Fields and Kuperberg, 2012; Porter et al., 2021), along with differential alpha and beta desynchronization (Subramaniam et al., 2019). Furthermore, during the subsequent delay period, we expected the IDC condition to elicit increased posterior alpha synchronization, reflecting the active shielding of internal thoughts from external interference (Kam et al., 2018; Mu and Han, 2010; O’Connell et al., 2009). Finally, our third objective was to utilize conditional quantile regression to uncover how these distinct neural and behavioral markers differentially influence working memory across the entire performance distribution.

## 2 Materials and methods

### 2.1 Participants

Thirty participants (16 females and 14 males, mean age = 25.2 years, SD = 2.3 years) participated in a study involving reporting perceptual behavior and simultaneous electroencephalogram (EEG) recordings. Participants were required to have a university degree with English as the medium of instruction. All the participants reported normal or corrected-to-normal vision, no history of color-blindness, were right-handed, and declared no history of neurological or psychiatric disorders. Participants were compensated monetarily for their time nominally and gave informed consent to participate in format approved by Institutional Human Ethics Committee (IHEC) of National Brain Research Centre (NBRC), India. EEG data from two participants were excluded because the electrode impedance exceeded the set threshold when checked at the end of the recording session. The sample size was determined based on practical constraints related to EEG data acquisition and participant availability, and a detailed *post hoc* sensitivity-based justification of this sample size is provided in Supplementary analysis S2.

### 2.2 Behavioral paradigm

**Figure 1:**
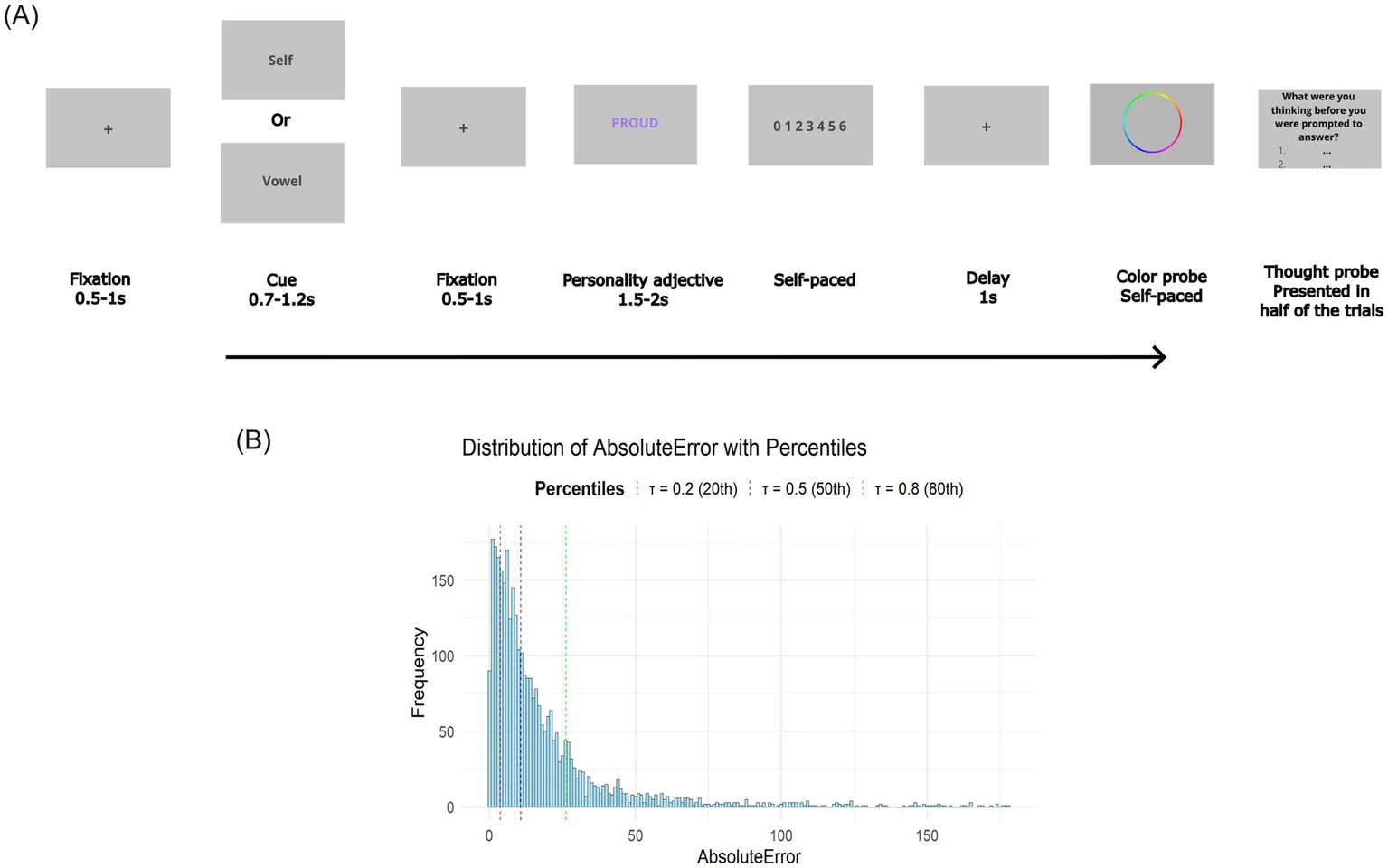
Behavioral paradigm and distribution of color-recall error. **(A)** Behavioral paradigm. Participants were given a cue, either ‘Self’ or ‘Vowel’ at the start of the trial. Then, a personality adjective was presented in a font color. The participant responded to the adjective according to the cue on a 7-point scale; if the cue was ‘self’, they respond to the question “How much this word describes your personality?,” and if the cue was ‘vowel’, they counted the number of vowels in the word. After a fixed-delay of 1 s, participant was probed for the memory of the font-color using a color wheel. In half of the trials randomly, a thought probe was presented at the end of the trial (see Methods section 2.2 for detail). **(B)** Distribution of color-recall error.

A complex color-recall working memory task was implemented (Figure 1A illustrates the trial sequence). At the start of each trial, a cue word appeared in the centre of the screen for a variable duration, randomly sampled from a discrete uniform duration between 0.7 s and 1.2 s ms in 0.1 s increment (mean = 0.97 s, SD = 0.17 s). The cue indicated which task the participant should perform:

- In the EDC condition, the cue word was *“vowel.”*
- In the IDC condition, the cue word was *“self.”*

Following the cue, a fixation cross was displayed for a randomly varied duration between 0.5 s and 1 s. Next, a personality adjective (see Supplementary Table 3) from a list of frequently used trait adjectives (Anderson, 1968) appeared in the centre of the screen, shown in a specific font color selected from hues of the color wheel (see section 2.3 for color-wheel design). Two distinct lists of adjectives were used for the IDC and EDC conditions. These lists were fixed across participants to meet task-specific constraints. For both conditions, adjectives were selected from Anderson’s (1968) normative set of personality trait adjectives, specifically choosing words with median likableness ratings to encourage self-reflection (Davey et al., 2016). For the EDC condition, to standardize response times and keep delays comparable across conditions (see Supplementary Figure 1), adjectives were selected to include between two and four vowels, based on pilot testing. Given that the lists were not counterbalanced, post-hoc analyses were conducted to compare them on key lexical properties including word frequency, length, and concreteness (see Supplementary analysis S1). The adjective was presented for a duration uniformly varied between 1.5 s and 2 s (mean = 1.78 s, SD = 0.16 s).

Depending on the cue, participants performed one of the two tasks:

- In EDC condition (vowel), participants counted the number of vowels in the word and responded on a seven-point scale indicating the count (0–6). This task was selected as a robust EDC control, as it requires sustained attention to the stimulus’s orthographic and perceptual features while minimizing engagement with its semantic content.
- In IDC condition (self), participants rated how well the adjective described their own personality on the seven-point scale, where 0 meant *“not at all me”* and 6 meant *“totally me.”* This SRP task was chosen as a canonical operationalization of IDC, designed to induce semantic and self-referential engagement, consistent with the frameworks of internally directed cognition (Dixon et al., 2014).”

After a fixed 1 s delay, a color wheel appeared on the screen to probe the font color of the adjective. Participants were instructed to respond as accurately and quickly as possible to both the rating and the color-recall task.

On 50% of the trials, selected randomly, a thought probe was presented after the color-recall response to assess the participant’s thought content during the delay period prior to color-recall (Huijser et al., 2018). The probe, adapted from prior studies (Huijser et al., 2018; Stawarczyk et al., 2011; Unsworth and Robison, 2016), asked: *“What were you thinking before you were prompted to answer?.”* Response options included: 1. *I tried to remember the color of the word*; 2. *I was still thinking about the word from the decision task*, 3. *I was evaluating aspects of the task*, 4. *I was distracted by the environment or my physical state*; 5. *I was daydreaming/thinking about task-unrelated matters*; 6. *I was not paying attention, but did not think about anything specific*. All the options were presented simultaneously to the participants, ensuring there was no cognitive load required to remember the choices.

### 2.3 Stimulus design and presentation

The experiment was designed in Matlab® (The MathWorks, Inc., Natick, MA, USA), using the Psychophysics Toolbox (Brainard, 1997) and displayed on a 22-inch LED monitor screen (75 Hz; 1,440 × 900 pixels) at a viewing distance of approximately 75 cm. The screen subtended a visual angle of 35.14° (width) and 22.40° (height). The stimuli were presented on a gray background (128, 128, 128; RGB255), and all text was generated in 50 pt. Arial font (approximately 1.7 cm letter height on screen). Therefore, each letter subtended approximately 1.3° of visual angle vertically and a 6–8 letter word occupied approximately 6–8° horizontally, making each word stimuli appear at the foveal-parafoveal range, eliminating the need for overt saccades or visual search across the screen. The color wheel was designed in RGB color space with hue angles mapped directly on the spatial angles (a total of 360 hues from 0° to 360°, with each hue incremented by 1°, inner radius = 6.44°, outer radius = 7.85°). The hue angle and spatial angle were kept constant throughout the experiment, so that each degree on the wheel uniquely corresponded to a specific hue. For each trial, the font color of the personality adjective was randomly selected from these 360 evenly spaced hues on the wheel (e.g., a hue of angle 0° correspond to red, 120° to green, and 240° to blue; see Figure 1). Participants completed 120 trials in total, consisting 60 trials per condition. The experiment was divided into three blocks of 40 trials each. There was a separate practice block of 12 trials with a different personality adjective list.

### 2.4 EEG data acquisition

EEG and behavioral data were acquired in a sound-attenuated room, and the ambient light was kept the same in all the recording sessions. EEG was acquired at a sampling frequency of 1 kHz using a 64-channel ActiChamp (Brain Products, Germany) with active electrodes for a better signal-to-noise ratio. The electrode placement used the 10% electrode placement system. The impedance was maintained below 15 kΩ and checked before and after the experiment. Electrode FCz was taken as a reference while recording the data. The Psychtoolbox was synchronized to the EEG acquisition system by sending triggers through parallel ports from the computer used to present stimuli to the EEG data acquisition computer.

### 2.5 Behavioral data analysis

Color recall accuracy was quantified by measuring the absolute angular error (absolute error), defined as the absolute difference between the presented hue and the hue reported by the participant. Reaction times for the intermediate task and color recall task were analysed. For thought-probe analysis, participant-wise percentage response of each option was calculated, and paired Wilcoxon signed rank test were conducted to compare percentage responses between the IDC and EDC conditions across the six options. Consistent with Huijser et al. (2018), the response option 1 was labelled as *on-task*, option 2 as *mental-elaboration*, option 3 as *task-related interference*, option 4 as *external distraction*, option 5 as *mind-wandering*, and option 6 as *inattentiveness*. All the responses, excluding *on-task* (option 1), were referred to jointly as *off-task*.

### 2.6 EEG data preprocessing and analysis

EEG data were analysed in Matlab® (The MathWorks, Inc., Natick, MA, USA) using custom scripts as well as EEGLAB (Delorme and Makeig, 2004) and ERPLAB (Lopez-Calderon and Luck, 2014) toolbox functions. First, the DC offset was removed, and the data were high-pass filtered at 0.1 Hz using a non-causal Butterworth filter. Data segments corresponding to break periods between trial blocks were removed. Channels exhibiting excessive noise—defined as prolonged flatlines (more than 5 s consecutively), high variance, or extreme amplitude fluctuations (greater than 1,000 μV)—were interpolated using spherical spline interpolation (Perrin et al., 1989; mean number of channels interpolated = 1.1).

To prepare the data for independent component analysis (ICA), segments exceeding a threshold of 500 μV within any 250 ms window (stepped every 50 ms) were removed. The data were then low-pass filtered at 45 Hz and down sampled to 250 Hz. ICA was performed using the infomax algorithm (*runica*), implemented in EEGLAB. Components were visually inspected, and those corresponding to ocular and muscular artifacts were removed (mean number of components removed = 9.6, SD = 4.7). The data were then re-referenced using the common-average re-referencing scheme.

The epochs corresponding to the encoding and delay periods were segmented for further analysis. The encoding epoch was defined from −500 ms to +1,200 ms relative to the onset of the personality adjective stimulus. The delay epoch spanned a fixed 1-s period immediately preceding the onset of the color recall wheel (see Figure 1A). Further, to reject commonly recorded artefactual potentials, which include skin potentials, movement artifacts, sudden voltage changes of unknown origin, the simple voltage threshold (SVT) and moving window peak-to-peak (MW) algorithm were used on the epoched data (Kappenman et al., 2021; Lopez-Calderon and Luck, 2014). A voltage threshold of −150 to 150 μV for SVT and a window size of 500 ms with a window step of 100 ms for MW was used. On average, 5.12% of the trials per participant were rejected.

Event-related potentials (ERP) were computed for the encoding epoch, and the pre-stimulus duration of 500 ms was used for baseline subtraction. The P200 window was defined as 152–252 ms post-stimulus, consistent with prior work on visual word recognition (Katyal et al., 2020; Schindler et al., 2023). For the late frontal positivity, an *a priori* time window of 500–800 ms was selected based on event-related potential literature on self-referential processing (Fields and Kuperberg, 2012; Mao et al., 2017; Nowicka et al., 2018). This temporal alignment is consistent with established time-course models of visual word recognition, which posit that deeper post-lexical semantic elaboration and integration processes occur only after initial lexical access is complete, typically emerging after 400 ms (Grainger and Holcomb, 2009). Statistical inference for ERP was carried out in two complementary ways. First, a non-parametric cluster-based permutation test, with FDR correction (*α* = 0.05), was used to identify the cluster of electrodes that were significantly different in both, the P200 and late frontal positivity windows. Following that, for hypothesis driven testing, mean amplitudes in the P200 and late frontal positivity windows from significantly different cluster of electrodes were compared using two-tailed paired-sample t-test.

Time-frequency analysis was performed using a complex Morlet wavelet transform across the 3–40 Hz range. The number of wavelet cycles increased linearly from 2 (at 3 Hz) to 12.5 (at 40 Hz), allowing for an optimal trade-off between temporal and frequency resolution. The resulting wavelet temporal window ranged from approximately 666 ms at 3 Hz to 313 ms at 40 Hz, power values were baseline-corrected by subtracting the average power in the pre-stimulus window (−400 to 0 ms) from post-stimulus power estimates. Based on prior works liking medial-frontal beta to self-generated thoughts (Subramaniam et al., 2019), and posterior alpha dynamics to internal and externally directed attentional states (Kam et al., 2018, 2022; O’Connell et al., 2009; Rihs et al., 2009), time-frequency analysis focused on medial-frontal and posterior parietal electrodes. The FieldTrip toolbox’s (Oostenveld et al., 2011) permutation testing, based on t-statistics computed at each time-frequency point and combined with cluster correction for multiple comparisons, was used to estimate significantly different time-frequency clusters between the conditions (Maris and Oostenveld, 2007). 10,000 permutations were computed, and the false positive (alpha) threshold was kept at 0.05 in all the statistical analyses.

### 2.7 Quantile regression

To study the relationship between EEG features, reaction times (RTs), and task-performance measured via recall error, conditional quantile regression was performed using *lqmm* package version 1.5.8 (Geraci, 2014) in R version 4.4.1.^1^ Participants’ color recall error showed a right-skewed distribution, with many small errors and fewer large deviations (see Figure 1B). Given that standard least-squares regression models the conditional mean, which is not a robust measure for skewed distributions, quantile regression was chosen to provide a more comprehensive analysis of the predictors across the entire performance distribution (Koenker and Bassett, 1978). To account for the variability in performance distribution, the models were estimated at three representative quantiles. *τ* = 0.5 was selected to model the conditional median, providing a robust estimate of the central tendency of the error distribution. To investigate the determinants of performance at the lower and upper ends of the distribution, symmetric quantiles of *τ* = 0.2 and *τ* = 0.8 were selected. This approach allowed for a specific examination of the factors influencing low error trials (i.e., those with small errors in the 20th percentile; *τ* = 0.2) and high error trials (i.e., those with large errors in the 80th percentile; *τ* = 0.8), respectively, providing a more nuanced understanding of how predictors may have differential effects on trials with varying levels of errors in color recall.

To identify the optimal set of predictors for recall error, four hierarchical quantile regression models of increasing complexity were constructed and compared. The selection of predictors was informed by the literature on internally directed cognition and self-referential processing (see introduction section). For medial-frontal beta (15–30 Hz), alpha (8–12 Hz), theta (4–7 Hz), and ERP (late frontal positivity), we averaged activity across a cluster comprising channels Fz, F1, F2, F3, F4, AF3, AF4, and AFz. For posterior alpha, and beta band activity, we averaged activity across a broad bilateral parieto-occipital cluster consisting of channels Pz, P1, P2, P3, P4, P5, P6, P7, P8, POz, PO3, PO4, PO7, PO8, Oz, O1, and O2. Each trial was treated as an independent observation, with SubjectID included as a random intercept to account for inter-subject variability.

- Model 1: Behavioral predictors and their interactions with condition type.

This model included only the condition type *(ConditionID)*, reaction times from the intermediate *(RT_I_)* as well as the color-recall task *(RT_C_)*, and their interaction with condition type as predictors.

- Model 2: Behavioral predictors, EEG features from encoding epoch, and their interactions with condition type.
$$\begin{array}{l} \mathrm{AbsoluteError}\left(\tau \right)=\beta 0+\beta 1\mathrm{ERP}+\beta 2{\mathrm{Beta}}_{\mathrm{encoding}}\\ +\beta 3{\mathrm{AlphaMF}}_{\mathrm{encoding}}+\beta 4{\mathrm{AlphaPO}}_{\mathrm{encoding}}+\beta 5{\mathrm{RT}}_{I}\\ +\beta 6{\mathrm{RT}}_{C}+\beta 7\mathrm{ConditionID}+\beta 8\left(\mathrm{ERP}\times \mathrm{ConditionID}\right)\\ +\beta 9\left({\mathrm{Beta}}_{\mathrm{encoding}}\times \mathrm{ConditionID}\right)+\beta 10\left({\mathrm{AlphaMF}}_{\mathrm{encoding}}\times \mathrm{ConditionID}\right)\\ +\beta 11\left({\mathrm{AlphaPO}}_{\mathrm{encoding}}\times \mathrm{ConditionID}\right)+\beta 12\left({\mathrm{RT}}_{I}\times \mathrm{ConditionID}\right)\\ +\beta 13\left({\mathrm{RT}}_{C}\times \mathrm{ConditionID}\right)+u+\varepsilon \tau \end{array}$$

u is the random intercept for each subject and ετ represents the residuals at each quantile τ.

- Model 3: Behavioral predictors, EEG features from the delay epoch, and their interaction with condition type.
- Model 4: Behavioral predictors, EEG features from encoding epoch and delay epoch, and their interactions with condition type

The model fit was compared using Akaike Information Criterion (AIC) and the model with the lowest AIC was selected as an optimal model and used for further analysis (Table 1). The *lqmm* package computed *p*-values using a block-bootstrap approach (Geraci, 2014; Geraci and Bottai, 2014), a robust method specifically chosen for its ability to handle clustered data in mixed models and to provide accurate inference without strong distributional assumptions. To account for multiple comparisons across predictors, the Benjamini-Hochberg False Discovery Rate (FDR) correction (Benjamini and Hochberg, 1995) was applied to the *p*-values. This approach controls the expected proportion of false positives while maintaining statistical power.

**Table 1:** Model comparison based on Akaike Information Criterion (AIC) values.

Model parameters	Model formula	AIC
Behavioral features only	m1 < − AbsoluteError ~ (RT_I + RT_C) * ConditionID	26,035.81
Behavioural and EEG features of encoding phase	m2 < − AbsoluteError ~ (RT_I + RT_C + ERP + Beta_encoding + AlphaMF_encoding + AlphaPO_encoding) * ConditionID	26,001.6
Behavioral and EEG features of delay phase	m3 < − AbsoluteError ~ (RT_I + RT_C + ERP + Theta_delay + MF_Beta_delay + PO_Beta_delay + Alpha_delay) * ConditionID	26,108.39
Behavioural and EEG features of encoding and delay phase	m4 < − AbsoluteError ~ (RT_I + RT_C + ERP + Beta_encoding + AlphaMF_encoding + AlphaPO_encoding +Theta_delay + MF_Beta_delay + PO_Beta_delay + Alpha_delay) * ConditionID	26,029.2

Lower AIC values indicate better fit. Model 1 included behavioral variables and their interactions with condition type; Model 2 and 3 included, in addition to behavioral features, EEG features of encoding and delay phases, respectively. Finally, Model 4 incorporated the behavioral features and the EEG features of the encoding and delay phases. MF - medial-frontal; PO-parieto-occipital; RT_I – reaction time in intermediate decision task; RT_C – reaction time in color-recall task.

## 3 Results

### 3.1 Participants reported more *off-task* in IDC

Participants’ responses to the thought-probe were analyzed to identify their brain state prior to the color-recall task. Since the thought probe required participants to report their mental states in six categories (see Methods section 2.2 for details), the percentage of responses to each option was subsequently calculated for each participant. Based on an earlier study (Huijser et al., 2018), different states of attention corresponding to each option was identified where option 1 was classified as *on-task*, option 2 as *mental elaboration*, option 3 as *task-related interference*, option 4 as *external distraction*, option 5 as *mind wandering*, and option 6 as *inattentiveness*. The distribution of responses between EDC and IDC for each option was then compared using paired non-parametric tests.

**Figure 2:**
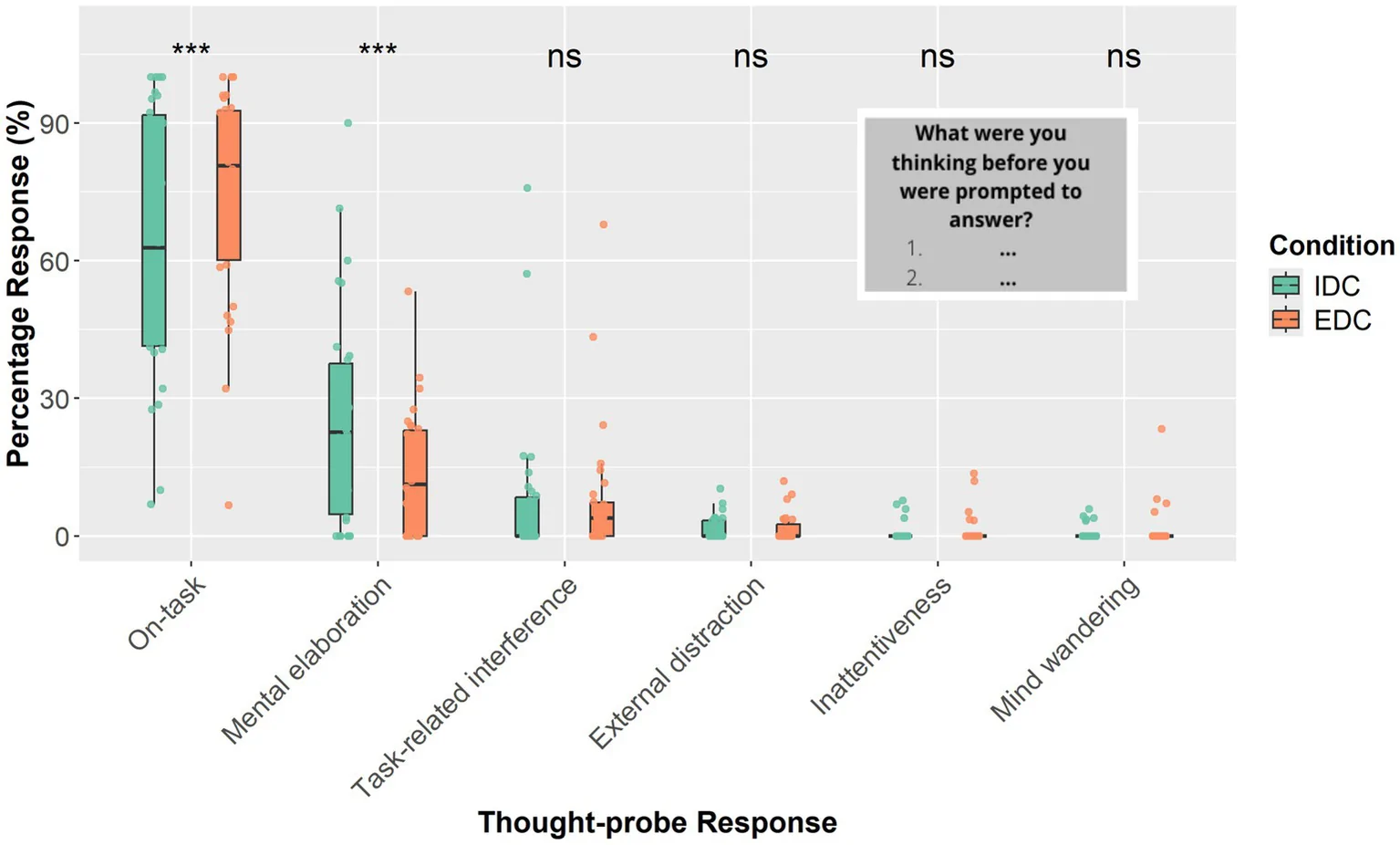
Response in the thought-probe plotted as percentage response for each option of the thought probe. Higher frequency of mental elaboration of the word and lower frequency of on-task in IDC relative to EDC.

The percentage of responses to the option 2, which is characterized as *Mental elaboration*, were significantly higher in IDC (Wilcoxon signed rank test, *Z* = 3.40, *p* = 0.0007, Cohen’s *d* = 0.67), whereas percentage of responses to option 1, characterized as *on-task.* Were lower in IDC relative to EDC (Wilcoxon signed rank test, *Z* = −3.33, *p* = 0.0009, Cohen’s *d* = −0.61) (Figure 2). The responses to *task-related interference, external distraction, inattentiveness*, and *mind wandering* were not significantly different between the IDC and EDC condition.

This self-report indicates a higher frequency on mental-elaboration of personality adjective during the delay period in IDC condition, supporting the validity of using the cue to manipulate the processing of word across conditions. Further this higher frequency of mental-elaboration could potentially hinder the rehearsal of the color to be recalled in the subsequent task, thereby impairing the performance in color-recall task.

### 3.2 Higher amplitude of late frontal positivity in IDC

**Figure 3:**
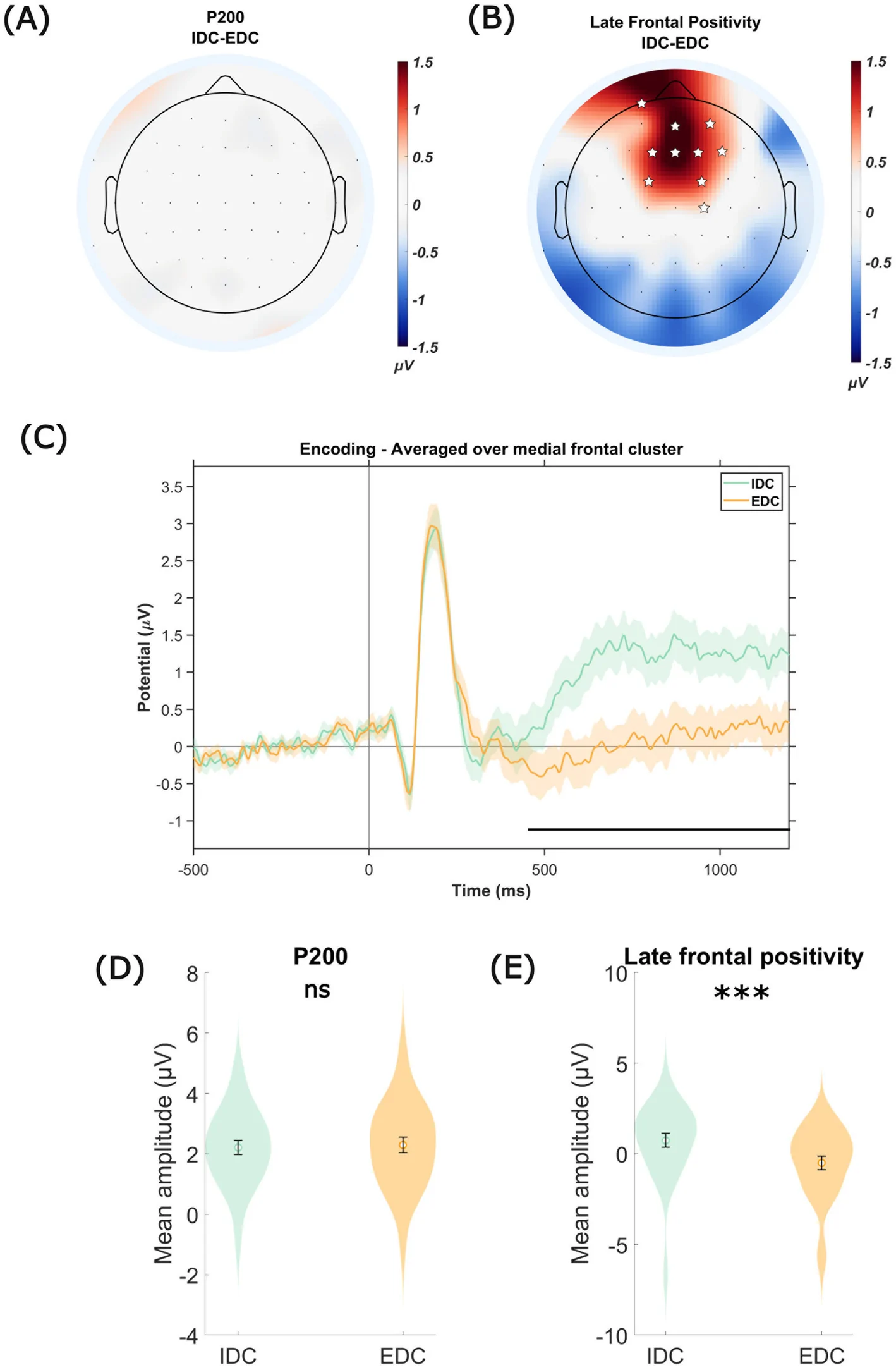
Event-related potentials during encoding in the IDC and EDC conditions. **(A)** Topographic difference map (IDC – EDC) for the P200 time window (152–252 ms). No electrodes showed significant condition differences. **(B)** Topographic difference map (IDC – EDC) for the late frontal positivity time window (500–800 ms). White stars indicate electrodes that showed significantly different amplitudes between conditions based on the cluster-based permutation test (*α* = 0.05, FDR-corrected). **(C)** Grand-average ERP waveforms during stimulus encoding. Waveform represent the average signal across the fronto-central cluster. Shaded regions around each waveform demote standard error. The solid black bar positioned along the *x*-axis highlights the time interval in which the IDC and EDC conditions differed significantly (permutation test, FDR corrected *α* = 0.05). **(D)** Mean amplitude of the P200 component at fronto-central cluster; no statistically significant difference was observed between conditions. **(E)** Mean amplitude of the late frontal positivity at fronto-central cluster, showing significantly greater positivity for IDC compared to EDC (***p < 0.001).

The scalp distribution of the instantaneous voltages was visualized at time points ranging from 0 ms to 1,000 ms, in 100 ms steps, following the onset of the word. This captured the temporal evolution and the sustained processing of the personality adjective over the scalp (see Supplementary Figure 2). These scalp maps showed that, in IDC condition, processing was predominantly sustained over frontal electrodes, whereas in the EDC condition, it was more pronounced over parietal electrodes. A non-parametric cluster-based permutation test (10,000 permutations; FRD-corrected at *α* = 0.05) revealed a fronto-central cluster of electrodes (Fz, F1, F2, F4, FC2, FC1, C2, AFz, AF4, and FP1) having significantly different amplitudes in the late frontal positivity window (500–800 ms) when compared between the conditions (Figure 3B). No electrodes showed significant differences between conditions in the P200 (152–252 ms) time window (Figure 3A).

Permutation based testing across the whole epoch revealed a temporal cluster emerging approximately after 450 ms post stimulus presentation and extending till the end of epoch (Figure 3C, the black bar along the *x*-axis highlights the significantly different temporal cluster). To quantify these differences, mean-amplitude tests for predefined temporal windows of the P200 (150–250 ms) and the late frontal positivity (500–800 ms) were conducted by averaging across this entire fronto-central spatial cluster. The mean amplitude of the late frontal positivity was significantly higher in the IDC condition compared to the EDC condition (*t*(27) = 5.92, *p* < 0.001, Cohen’s *d* = 1.12; Figure 3E). This enhanced frontal late positivity can be interpreted as a neural signature of attentional capture by self-referential content. This distinguishes the deeper cognitive evaluation of the adjective in the IDC condition from the more perceptual analysis required in the EDC condition. Conversely, for the P200 component, no significant difference between the IDC and EDC conditions was observed across the frontal cluster (*t*(27) = −0.86, *p* = 0.4, Cohen’s *d* = −0.16; Figure 3D), indicating that early-stage perceptual processing of the stimulus was not distinct between the two conditions.

### 3.3 Spectral differences in IDC relative to EDC

Next, time-frequency analysis was performed on the EEG data to examine differences in spectral power between the two conditions. Specifically, spectral features were analyzed during two time-windows: the encoding period and the delay period immediately preceding the color-recall task. A complex Morlet wavelet transform was applied to perform time-frequency analysis across frequencies 3–40 Hz.

**Figure 4:**
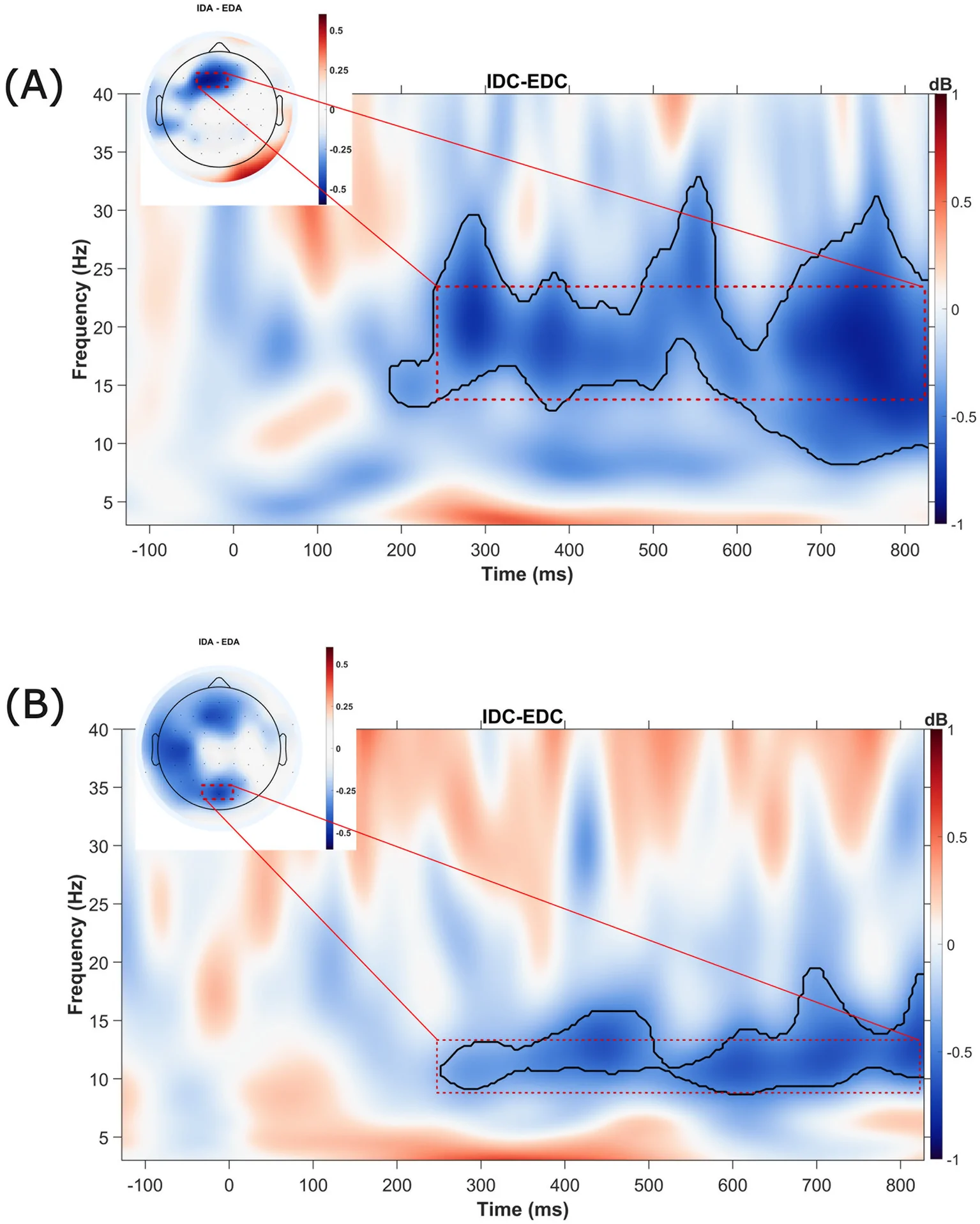
Time-frequency plots showing difference in power during encoding of the stimulus (IDC minus EDC). **(A)** The difference in power between IDC and EDC over medial frontal sensors (F1 plotted for representation) and **(B)** posterior sensors (POz plotted for representation). Insets show topographical distribution of the condition difference with electrode locations marked. Black contours outline cluster of significant difference (*p* < 0.05, corrected).

In the encoding epoch, a cluster-based permutation test based on t-statistics indicated a significant effect of condition (*p* < 0.05). In the observed data, this corresponded to a cluster showing higher event-related desynchronization (ERD) in beta frequency band activity in IDC compared to EDC over the medial-frontal sensors (F1 plotted for representation; see Figure 4A). Further, a significantly higher event-related desynchronization (ERD) was observed in the alpha frequency band over parieto-occipital sensors (POz plotted for representation; see Figure 4B) and medial-frontal sensors in the IDC condition (see Supplementary Figure 3 for time-frequency plots of each condition).

**Figure 5:**
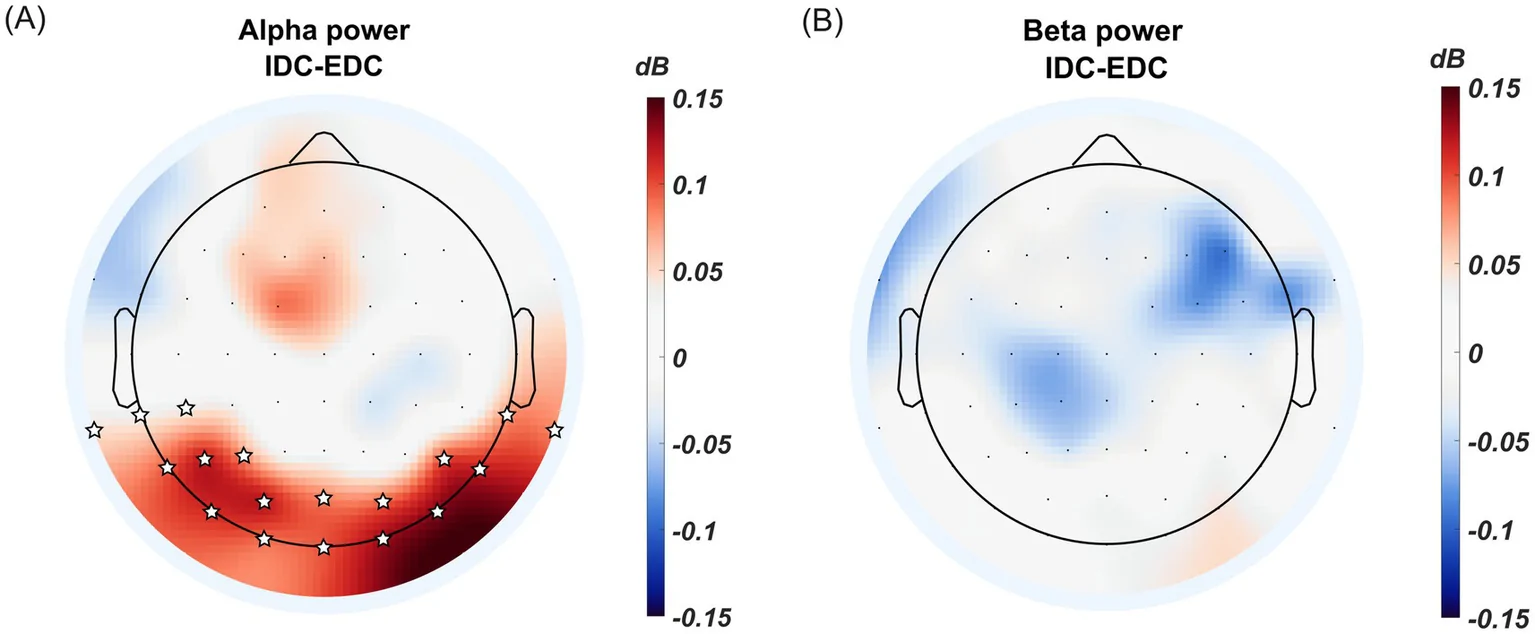
Topography plot of difference in power between IDC and EDC in **(A)** alpha band (8–12 Hz) and **(B)** beta band (15–30 Hz) during the delay period prior to the color-recall. White stars indicate statistically significant sensors (*α* = 0.05, FDR-corrected) based on the cluster-based permutation.

Additionally, during the delay period prior to the recall of the color, a higher alpha power was observed in IDC over occipito-parietal sensors (Oz, O1, O2, PO7, PO8, P7, P8, P5, P3, PO3, POZ, PO4, P6, CP5, TP7, TP8, TP9) relative to the EDC condition (Figure 5A). No significant difference was observed between the two conditions in beta band power in delay period (Figure 5B).

### 3.4 Effect of condition type on color-recall accuracy

Overall performance on the color-recall task exhibited a strong rightward skew (Figure 1B). Subject-level summary statistics revealed that the macroscopic absolute angular error in the IDC condition (Mean = 18.26°, Median = 11.92°, SD = 20.62°) was not significantly different from the EDC condition (Mean = 19.22°, Median = 11.99°, SD = 22.51°); *t*(27) = −0.84, *p* = 0.40. As evidenced by the substantial positive divergence of the mean from the median, as well as an inflated standard deviation that exceeds the mean itself, the arithmetic mean is disproportionately dragged by a long right tail of trials with high angular error. Because these summary statistics fail to accurately represent the central tendency or dynamic trial-by-trial variance of skewed data, we utilized non-parametric linear quantile mixed models (LQMM) for our primary analysis.

To investigate how neural and behavioral predictors influence absolute error across different levels of performance, LQMM were fitted at three quantiles: *τ* = 0.2 (lower errors), *τ* = 0.5 (median errors), and *τ* = 0.8 (higher errors) (see Methods section 2.7 for details). The condition type (IDC or EDC) and RTs in both the intermediate and color-recall task were added as fixed (see Model 2, Methods section 2.7). It also incorporated interaction between RTs with condition type.

**Figure 6:**
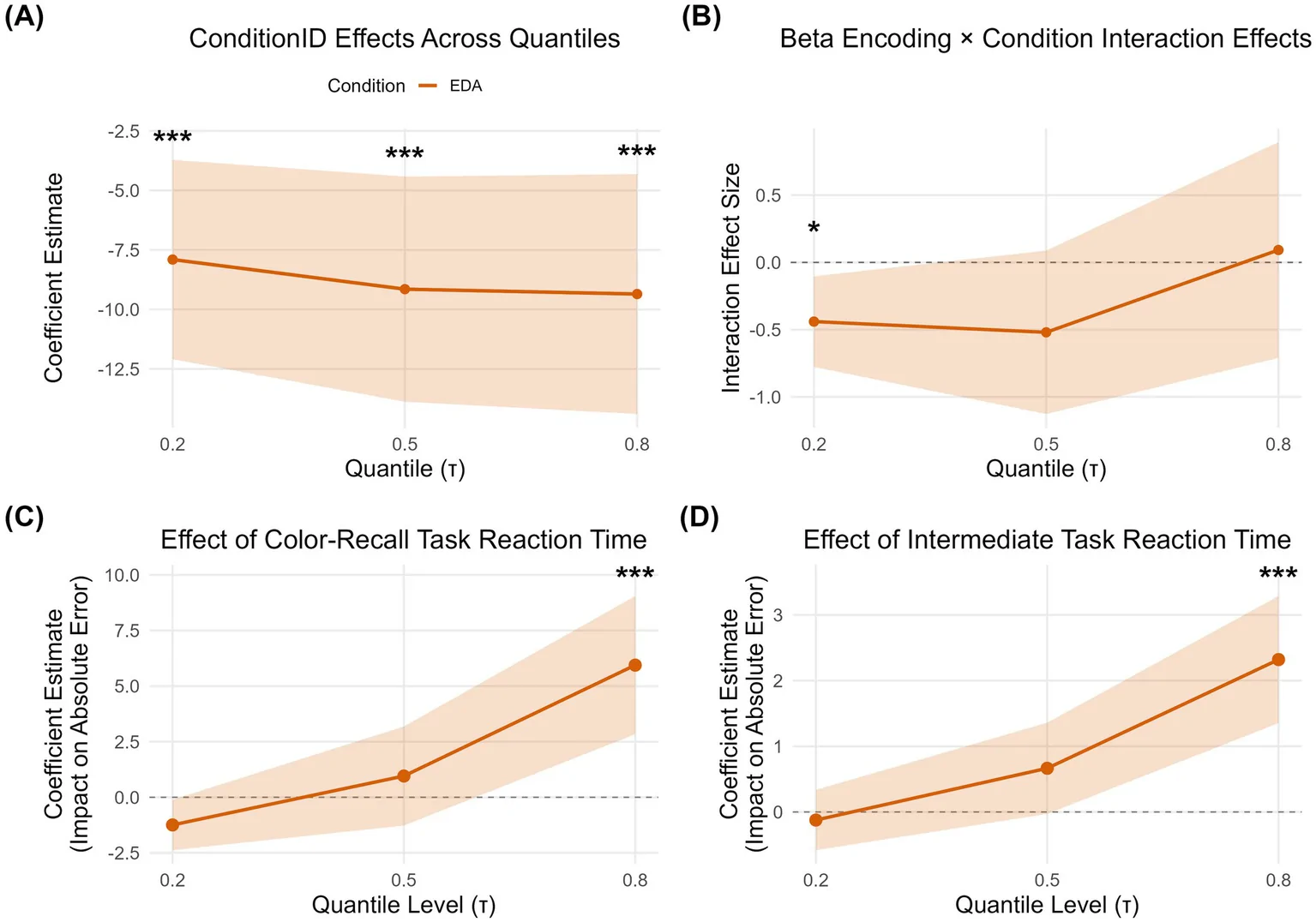
Effect of neural and behavioral predictors across quantiles of error response. **(A)** Estimated coefficients for the main effect of ConditionID (EDC) across quantiles. **(B)** Interaction effect between beta power during encoding and condition (EDC vs. IDC) across quantiles. **(C)** Main effect of reaction time in color recall task on error in color recall. **(D)** Main effect of reaction time in intermediate task on color recall. The shaded area represents the 95% confidence interval. Asterisks denote statistically significant effect (FDR corrected).

Fixed effect of ConditionID revealed that participants made fewer errors in EDC relative to the IDC condition across all quantiles of the error distribution (Figure 6A). Interestingly, this effect becomes much stronger from lower quantiles (*τ* = 0.2, *β* = −7.90, SE = 2.13, *p* = 0.0011) to median and upper quantiles (*τ* = 0.5, *β* = −9.15, SE = 2.41, *p* = 0.001; *τ* = 0.8, *β* = −9.35, SE = 2.57, *p* = 0.0013; see Table 2 for the parameter estimates, standard errors (SE), and FDR corrected *p*-values). Reaction times did not have any meaningful correlations with performance in color recall in lower quantile, i.e., *τ* = 0.2 (RT_I_, *β* = −0.12, SE = 0.23, *p* = 0.76; RT_C_, *β* = −1.24, SE = 0.57, *p* = 0.09) and median quantile, i.e., *τ* = 0.5 (RT_I_, *β* = 0.66, SE = 0.35, *p* = 0.17; RT_C_, *β* = 0.95, SE = 1.13, *p* = 0.6) trials. However, slower responses in both the intermediate task and color recall task (RT_I_, *β* = 2.32, SE = 0.49, *p* < 0.001; RT_C_, *β* = 5.94, SE = 1.58, p = 0.001) correlated with higher errors (*τ* = 0.8) irrespective of the conditions (Figures 6C,D).

**Table 2:** Quantile regression results across three quantile levels (*τ* = 0.2, 0.5, 0.8).

*τ*	Coefficient	Estimate	Std. error	*p*-value	FDR adjusted *p*
0.2	(Intercept)	7.3633	1.9432	0.0002	0.001
	RT_I_	−0.1231	0.2337	0.5984	0.7617
	RT_C_	−1.2443	0.5783	0.0319	0.0957
	ERP	0.0174	0.0388	0.6546	0.7676
	Beta_encoding_	0.2086	0.1155	0.0715	0.1876
	AlphaMF_encoding_	−0.0185	0.0666	0.7811	0.8633
	AlphaPO_encoding_	0.0087	0.1034	0.9327	0.9555
	**ConditionID**	**−7.9065**	**2.1361**	**0.0002**	**0.0011**
	**RT**_**I**_ **: ConditionID**	**0.8345**	**0.308**	**0.007**	**0.0244**
	**RT**_**C**_ **: ConditionID**	**2.7601**	**0.7989**	**0.0006**	**0.0023**
	ERP: ConditionID	−0.0147	0.0477	0.758	0.8604
	**Beta**_**encoding**_ **: ConditionID**	**−0.4401**	**0.1718**	**0.0107**	**0.0346**
	AlphaMF_encoding_: ConditionID	0.0761	0.0934	0.4157	0.602
	AlphaPO_encoding_: ConditionID	−0.1263	0.1366	0.3555	0.5742
0.5	(Intercept)	9.5116	2.2082	0	0.0002
	RT_I_	0.6652	0.3541	0.0609	0.1705
	RT_C_	0.9558	1.1355	0.4003	0.6005
	ERP	0.0294	0.0665	0.6579	0.7676
	Beta_encoding_	0.1256	0.1748	0.4727	0.6204
	AlphaMF_encoding_	−0.1317	0.1368	0.3362	0.5708
	AlphaPO_encoding_	0.0817	0.1743	0.6392	0.7676
	**ConditionID**	**−9.1501**	**2.4134**	**0.0002**	**0.001**
	RT_I_: ConditionID	0.5203	0.3807	0.1724	0.3621
	**RT**_**C**_ **: ConditionID**	**4.1401**	**1.0908**	**0.0002**	**0.001**
	ERP: ConditionID	−0.0689	0.0798	0.3885	0.6005
	Beta_encoding_: ConditionID	−0.5199	0.3099	0.0941	0.2324
	AlphaMF_encoding_: ConditionID	0.2318	0.1746	0.1849	0.3698
	AlphaPO_encoding_: ConditionID	−0.3466	0.216	0.1092	0.2549
0.8	(Intercept)	11.5974	2.3309	0	0
	**RT**_**I**_	**2.3201**	**0.4932**	**0**	**0**
	**RT**_**C**_	**5.9421**	**1.5847**	**0.0002**	**0.001**
	ERP	0.0129	0.1115	0.908	0.9534
	Beta_encoding_	−0.2584	0.337	0.4436	0.6204
	AlphaMF_encoding_	0.01	0.2516	0.9683	0.9683
	AlphaPO_encoding_	−0.3297	0.3157	0.2968	0.5667
	**ConditionID**	**−9.3549**	**2.5718**	**0.0003**	**0.0013**
	RT_I_: ConditionID	0.5258	0.5312	0.3228	0.5708
	**RT**_**C**_ **: ConditionID**	**6.2737**	**1.1447**	**0**	**0**
	ERP: ConditionID	0.142	0.1486	0.3398	0.5708
	Beta_encoding_: ConditionID	0.0923	0.4092	0.8216	0.8848
	AlphaMF_encoding_: ConditionID	0.3908	0.2745	0.1551	0.3429
	AlphaPO_encoding_: ConditionID	−0.3024	0.4073	0.4582	0.6204

The models include behavioral predictors (reaction times from intermediate [RT_I_] and color-recall [RT_C_] tasks), EEG features from the encoding epoch (late frontal positivity amplitude, beta and alpha power at medial-frontal and posterior electrodes), condition type (ConditionID), and interaction terms between ConditionID and all predictors. Bold rows are statistically significant. AlphaMF – Alpha band activity over medial frontal sensors; AlphaPO – Alpha band activity over parieto-occipital sensors.

### 3.5 Beta power over medial-frontal sensors during encoding is a significant modulator in color-recall performance

To identify the optimal model for predicting absolute error, a series of quantile regression models of increasing complexity were compared using AIC (Table 1). First, simpler models were tested with only RTs or condition type (IDC vs. EDC) as predictors, followed by models that also included EEG features from the encoding and delay periods. Based on lowest AIC, the final model included late frontal positivity amplitude, medial-frontal beta and alpha during encoding, parietal-occipital alpha during encoding, RTs from the intermediate and color-recall tasks, and ConditionID as fixed effects. It also incorporated interactions between each predictor and ConditionID, as well as a random intercept for SubjectID (see Model 2 in Methods section 2.7). Table 2 summarizes the parameter estimates, standard errors (SE), and FDR corrected p-values for each predictor at each quantile.

Quantile regression revealed a significant interaction between medial-frontal beta power and ConditionID. The relationship between medial-frontal encoding beta power and color-recall error was dependent on the condition type at the lower error quantile (*τ* = 0.2; *β* = −0.44, SE = 0.17, *p* = 0.035) (see Figure 6B). To break down this interaction, we examined the effect of beta power within each condition separately. At this lower quantile (*τ* = 0.2), the effect of beta power in the IDC condition was non-significant (*β* = 0.21, *p* = 0.18), whereas the relationship in the EDC condition was significantly negative (simple slope: *β* = −0.23), indicating that higher beta power was associated with lower errors in color-recall. No significant interaction was observed at the median (*τ* = 0.5) or upper (*τ* = 0.8) quantiles. This indicates that the beneficial role of beta power in reducing recall error was present only during EDC and was disrupted during the IDC. Other EEG measures, including late frontal positivity amplitude and alpha power, did not meaningfully affect the color recall (see Table 2). Furthermore, a significant interaction between color-recall RT and ConditionID was present across all quantiles; specifically, in the EDC condition, longer color-recall RTs were correlated with color-recall errors (*τ* = 0.2: *β* = 2.76, SE = 0.80, *p* = 0.002; *τ* = 0.5: *β* = 4.14, SE = 1.09, *p* = 0.001; *τ* = 0.8: *β* = 6.27, SE = 1.14, *p* < 0.001), suggesting a shared variance with the color-recall performance.

## 4 Discussion

This study investigated the effects of internally directed cognition (IDC) on working memory and the underlying neural mechanisms associated with such effects. A self-referential processing task was employed with a color recall working memory paradigm. In contrast, for externally directed cognition (EDC) condition, a vowel-counting task was used instead of the self-referential task. The main empirical findings of the study were as follows, *First*, behaviorally, it was found that participants had higher accuracy in color recall in EDC condition compared to the IDC condition across all levels of performance. Additionally, participants’ color recall error response followed a right-skewed distribution, with many small errors and fewer large errors. *Second*, late frontal positivity was observed over medial frontal sensors during stimulus encoding in the IDC condition, suggesting self-referential engagement with the personality adjectives. Additionally, event-related desynchronization (ERD) in the alpha and beta bands over medial-frontal sensors, and in alpha band over posterior sensors was greater in IDC relative to the EDC condition during encoding. During the delay period, higher alpha power was observed in IDC over occipito-parietal sensors compared to the EDC condition. *Third*, medial-frontal beta power in encoding, in interaction with condition type, significantly influenced color-recall accuracy at lower quantiles of error distribution, whereas responses in high quantiles were significantly correlated with reaction times.

Prior studies have shown that self-referential processing triggers self-generated thoughts (Huijser et al., 2018), which are considered a core component of IDC (Dixon et al., 2014). This paradigm, therefore, provides a robust approach to examining IDC mechanisms. The present thought-probe analyses revealed that participants were more off-task during the delay period in the IDC condition (Figure 2), evidenced by a higher frequency of mental elaboration. Consistent with these findings, color-recall performance was significantly poorer in IDC trials compared to EDC trials. These results suggest that self-referential processing may divert cognitive resources from the rehearsal of perceptual features, thereby impairing task performance.

Electrophysiological analyses confirmed that the personality adjectives were processed differently across conditions. Previous research on self-referential processing using EEG has shown distinct ERP signatures, frequently reporting enhanced late positivities in response to personality adjectives evaluated for self-relevance (Fields and Kuperberg, 2012; Kotlewska and Nowicka, 2016; Magno and Allan, 2007; Mao et al., 2017; Nowicka et al., 2018; Porter et al., 2021). In the present paradigm, processing the personality adjective in a self-referential manner, as cued at the beginning of the trial, led to a significantly higher amplitude in a late frontal positivity relative to counting the number of vowels in the word (Figure 3C). While canonical, posteriorly distributed late positive potentials are typically linked to sustained emotional engagement (Hajcak and Foti, 2020), frontally distributed late positivities are specifically elicited by tasks demanding the active evaluation and retrieval of self-related information (Fields and Kuperberg, 2012; Mao et al., 2017). Within the context of the current paradigm, this enhanced frontal late positivity serves as a neural signature of attentional capture by self-referential content. It reflects the elaborative cognitive engagement required to evaluate an adjective in relation to one’s self-concept. This elaborative engagement is distinct from the perceptual analysis required by the EDC task, suggesting that the late frontal positivity effect in this study indexes the specific cognitive operations inherent to self-referential judgment. The P200 component in ERP, which is reported to be responsible for exogenous attentional capture (Carretié, 2014; Kanske et al., 2011) and automatic semantic processing of the word (Crowley and Colrain, 2004; Lei et al., 2017; Schindler et al., 2023), was not different between conditions suggesting that the early-stage processing might not differ between conditions. Further, the observed differences in late frontal positivities components during the processing of the personality adjective align with the time-course model proposed by Grainger and Holcomb (2009), which suggests that semantic elaboration and integration processes occur after 400 ms of stimulus presentation, corresponding to the time window in which the late frontal positivities differences were observed in this study. Thus, taking the current electrophysiological results and the literature into account, one can concur that the personality adjective undergoes deeper self-referential processing in the IDC condition compared to the EDC condition.

Time-frequency analyses revealed a greater alpha desynchronization during the encoding of personality adjectives in the IDC condition over both medial-frontal and parietal sensors (Figure 4). These observations further strengthen the inference in the literature that alpha desynchronization correlates with emotional engagement with the stimuli (De Cesarei and Codispoti, 2011; Knyazev et al., 2008; Otten and Jonas, 2014; Schubring and Schupp, 2019). Conversely, in the delay period prior to color-recall, higher alpha power over posterior sensors was observed in IDC (Figure 5A). This alpha increase in the delay period is a signature of internally directed attention (Kam et al., 2018; O’Connell et al., 2009). Taking together, the thought-probe results, ERP analyses, and the time-frequency findings indicate that the IDC condition promotes semantic and self-referential engagement during stimulus encoding and internal focus during the delay period, making this paradigm a robust choice to study IDC mechanisms. However, one caveat of the present design is that, although IDC is examined in contrast to EDC, certain cognitive systems such as those involved in goal maintenance and working memory are engaged in both conditions, making it challenging to unequivocally isolate neural processes purely specific to IDC.

Quantile regression was employed to assess the differential contributions of ERP, beta, and alpha power, and reaction times under internal and externally directed cognition conditions. This is because the traditional regression methods, such as ordinary least squares, estimate the mean effect of the predictors on an outcome variable assuming a constant relationship across all levels of the dependent variable. However, in present study, given that the distribution of color-recall error is not homogenous across its range, the quantile regression examined how the independent variables predict different parts of the error distribution, thus allowing us to investigate effects at different levels of task performance (lower, median, and high errors in color-recall, corresponding to lower, median and upper quantile respectively). The effect of condition type (IDC vs. EDC) was significant across all quantiles, indicating a consistent performance advantage in the EDC condition. However, the magnitude of this effect increased at median and upper quantiles, suggesting that while the condition difference was already present in low-error trials, it became even more pronounced in trials with higher error in color-recall. While group-level differences in alpha and ERP measures were evident between the conditions, they did not significantly predict performance variance across individual trial observations. A possible explanation could be that these measures reflect neural processes that are not directly linked to performance in the color-recall task in the current paradigm but are just the signatures of internally directed attention.

The IDC condition likely requires deeper semantic and self-referential processing. This leads to more self-generated thoughts in IDC compared to EDC. The activation of medial prefrontal cortex (mPFC) is reported in both generation and retrieval of self-generated information (Subramaniam et al., 2012, 2019; Vinogradov et al., 2008). Magnetoencephalography (MEG) study by Subramaniam et al. (2019) further reported the suppression of mPFC beta power during self-generated information. In line with the previous findings, a reduction of beta power over medial-frontal sensors in IDC relative to EDC in the encoding epoch was observed in the present study. Further, our quantile regression analysis revealed that the extent of beta power change at frontal sensors had a significant, condition-dependent impact on color-recall. Taken together, more self-generated thoughts in IDC, as evidenced by thought-probe (Figure 2) and neural signatures (Figure 4), could lead to poor performance on color-recall. This may occur through two, not necessarily exclusive, mechanisms. One possibility is the more self-generated thoughts during the encoding phase of the trial in IDC could lead to cortical information processing facilitated by alpha and beta desynchronization (Hanslmayr et al., 2012). However, this reduces the resources for encoding the low-priority (font color) perceptual features and thus affects their recall. Alternatively, the shift toward internally directed cognition during the delay period was indexed by a concomitant increase in parieto-occipital alpha activity (Figure 5A). In line with the inhibition by alpha oscillations framework (Klimesch et al., 2007), this elevated posterior alpha is a signature of internal attention, reflecting the active suppression of visual cortices to shield internal thought from external sensory interference (Benedek et al., 2014, 2016; Kam et al., 2018, 2021). This active suppression of the visual cortex should directly conflict with the maintenance of a visual color representation, leading to poor rehearsal and thus poor performance in color-recall task. These mechanisms are not mutually exclusive, and IDC could plausibly interfere with both encoding and maintenance-related processes. However, since the time-frequency parameters of the delay period did not improve the quantile regression model fit, and the medial-frontal beta power during encoding did influence the trial-by-trial performance (Figure 6B), the current study provides evidence in support of the former mechanism.

In conclusion, the predominance of low error trials (see Figure 1B) indicate that the participants performed relatively well on the color recall task. Consequently, the quantile regression analyses reveal that medial-frontal beta power in interaction with condition type during encoding significantly predicts performance at these quantiles. Conversely, reaction times became dominant predictors only in the high-error trials, which are relatively few, indicating a shift to speed-accuracy tradeoff in such trials. Moreover, by integrating trial-level EEG with quantile regression, our findings indicate that the association between beta power and performance is dependent on the attentional state during encoding. Specifically, the inverse relation of beta power and error in color-recall observed in the EDC condition was not evident during the IDC condition. With this dissociation in place, we speculate that the IDC state may interfere with the neural processes that support effective perceptual binding. The observed distinct spectral mechanisms across task phases predict how IDC might impacts visual working memory processing during goal-directed tasks.

## 5 Limitations of the present study

A potential limitation of the present study is the absence of eye movement monitoring. It could be argued that performance differences in color-recall between the IDC and EDC conditions might stem from differences in basic visual encoding, such as fewer fixations on the word stimulus during the IDC condition. However, given that all word stimuli were presented centrally at the foveal-parafoveal range on a uniform background, and participants were instructed to maintain a fixed viewing distance, the opportunity for differential visual exposure was minimal. An alternative hypothesis, grounded in the oculomotor literature, is that the distinct cognitive demands of our tasks would elicit different functional gaze strategies. The perceptual vowel-counting task (EDC) would likely encourage active scanning (i.e., more, shorter fixations), whereas the introspective self-referential task (IDC) would likely induce a state of “perceptual decoupling” to shield internal processing from external distraction (Benedek et al., 2017). This state is often characterized by fewer and longer fixations, reflecting a functional shift of attention inward. Nevertheless, without direct oculomotor data, this remains an interpretation. Future research combining EEG with eye-tracking would be invaluable for directly testing these competing hypotheses regarding gaze behavior during internal versus external attention. We also acknowledge that our use of absolute angular error assumes a linear mapping between the physical stimulus space and psychophysical color space, a limitation highlighted in Schurgin et al. (2020). However, because this non-linearity applies equally to both attentional conditions, the observed relative difference in performance between them remains a valid indicator of the cognitive cost imposed by the IDC task. Another potential limitation of the current study is the use of two distinct, non-counterbalanced lists of adjectives for the IDC and EDC conditions. This design choice was necessitated by the specific constraints of each task; however, it means that items were nested within conditions, raising the possibility that observed differences could be driven by lexical properties of the stimuli rather than the attentional manipulation. We sought to mitigate this concern by matching the lists on word frequency, concreteness, and emotional valence Furthermore, while word length necessarily differed between lists, we performed a control analysis by re-estimating our primary quantile mixed-effects model with word length included as a covariate. The results of this analysis, detailed in Supplementary material, confirmed that word length did not significantly predict color-recall performance, and all critical effects of condition and beta power remained unchanged. Nevertheless, we acknowledge this design constraint, and future studies could aim to develop paradigms that allow for full counterbalancing of stimuli. Finally, a limitation of the present study is the inability to perform additional analyses restricting our models to specific subjective cognitive states. Because thought probes were only presented on half of the trials to maintain task flow, filtering the dataset by subjective responses would discard the majority of the data. Future research employing higher trial counts or continuous state-tracking will be necessary to decouple these state-dependent neural dynamics.

## Data Availability

The raw EEG data used in this study is available from the corresponding authors upon reasonable request. However, the processed EEG data and all the relevant codes used for subsequent analysis in this study are available in the provided link https://drive.google.com/drive/u/0/folders/19X5xdNLVueJf5NniAL4JtUKgXewgqWEH.

## Footnotes

1
http://www.R-project.org
