# Correction: Differential role of beta band activity in a dual-task working memory paradigm under internally vs. externally directed cognition

**DOI:** 10.3389/fnhum.2026.1937421

**Published:** 2026-09-16

**Authors:** Ankit Yadav, Arpan Banerjee, Dipanjan Roy

**Affiliations:** 1 National Brain Research Centre, Gurugram, India; 2 Indian Institute of Technology, Jodhpur, India

**Keywords:** beta band activity, dual-task paradigm, internally directed cognition, quantile regression, working memory

Author Arpan Banerjee was erroneously omitted as corresponding author. The correct corresponding authors are Ankit Yadav (ankit.b19@nbrc.ac.in), Arpan Banerjee (arpan@nbrc.ac.in), and Dipanjan Roy (droy@iitj.ac.in).

The original version of this article has been updated.

