# Supplementary material for: Differential role of beta band activity in a dual-task working memory paradigm under internally vs. externally directed cognition
Source: Front Hum Neurosci. 2026 May 12;20:1791453. doi: 10.3389/fnhum.2026.1791453 (PMC13201523; doi:10.3389/fnhum.2026.1791453)
Supplement: Supplementary file 1 [file Data_Sheet_1.docx]

**Supplementary data and analysis**

**S1. Control analysis for lexical features of word lists**

The lexical analysis was conducted to compare the two lists (**Supplementary table 3**). The lists were compared on word frequency, word length (characters per adjective), and concreteness. Based on a list published by Brysbaert and New, 2009, word frequency per million was computed and compared between the two list. No significant difference was observed in word frequency (two sample t-test, t = -1.81, p = 0.07). Concreteness, defined as the degree to which the concept denoted by a word refers to a perceptible entity, was computed using concreteness ratings published in Brysbaert et al., 2014. No significant difference was observed in concreteness ratings between the two lists (two sample t-test, t = -0.71, p = 0.47).

The adjective list did differ significantly in mean word length (two sample t-test, t = 5.2, p < 0.05). This was expected as we constrained the personality adjectives used in EDC condition to contain two to four number of vowels, matched in valence to the adjectives in IDC. To mitigate this confound and to ensure that the word length did not influence the color-recall behavior, we re-estimated the quantile mixed-effects model including word length as a fixed covariate along with all other predictors and their interactions with condition:

$$\mathrm{AbsoluteError}\left( \tau\right)=\beta0+\beta1LPP+\beta2\mathrm{Beta}_{encoding}+\beta3AlphaF1_{encoding}+\beta4\mathrm{AlphaPOz}_{encoding}+\beta5\mathrm{RT}_{I}+\beta6\mathrm{RT}_{C}+\beta7WordLenght+\beta8ConditionID+\beta9\left( LPP\times ConditionID \right)+\beta10\left( \mathrm{Beta}_{encoding}\times ConditionID \right)+\beta11\left( \mathrm{AlphaF}1_{\mathrm{encoding}}\times ConditionID \right)+\beta12\left( \mathrm{AlphaPOz}_{\mathrm{encoding}}\times ConditionID \right)+\beta13\left( \mathrm{RT}_{I}\times ConditionID \right)+\beta14\left( \mathrm{RT}_{C}\times ConditionID \right)+u+\varepsilon\tau$$

Across all quantiles (τ = 0.2, 0.5, 0.8), **word length showed no significant effect on color-recall performance** (τ = 0.2, β = -0.14, SE = 0.07, p = 0.18; τ = 0.5, β = 0.12, SE = 0.14, p = 0.55; τ = 0.8, β = 0.28, SE = 0.20, p = 0.36; **Supplementary table 4**). Further, the main effect of condition type (IDC vs. EDC) and the critical interaction effect of beta power and condition type remained unchanged, with the same direction and magnitude as in the main analysis (**see Table 2 and Supplementary table 4**). This indicate that the difference in word length did not drive the changes in color-recall behavior.

**S2. Sensitivity power analysis**

To verify the adequacy of sample size post hoc, simulation-based (Kumle et al., 2021) sensitivity power analysis was conducted using the parameters estimated from our linear quantile mixed model (LQMM) (Model 2; Methods section 2.7). Using the coefficients, subject-level variability, and residual dispersion estimated from the original dataset, 1,000 simulated datasets were generated to replicate the structure of the original experiment in terms of subjects (N = 28), trials, and conditions. For each simulated dataset, the same linear quantile mixed model was re-fitted, and the statistical significance (p < 0.05) of the target effects was evaluated. The main effect of Condition type (IDC vs. EDC) and the interaction between beta power and Condition type were both examined. To evaluate sensitivity, the simulations were repeated after reducing the effect size to 75% and 50% of the originally observed magnitude, thereby assessing how the sample size performed under smaller effect scenarios. Statistical power was computed as the proportion of simulations in which the target effect was detected as significant (p < 0.05) out of the total number of successful model fits, providing an empirical estimate of the likelihood of detecting true effects of similar or smaller magnitudes given the study’s sample size and data structure.

For condition type:

**Supplementary table 1** Results from sensitivity power analysis for the effect of Condition type (IDC vs EDC) at a varying effect size multiplier.

| **Effect size multiplier** | **Power for effect of Condition type** | **tau** |
| --- | --- | --- |
| Observed effect X 1 | 1 | 0.2 |
| Observed effect X 0.75 | 0.997 | 0.2 |
| Observed effect X 0.5 | 0.905 | 0.2 |
|  |  |  |
| Observed effect X 1 | 1 | 0.5 |
| Observed effect X 0.75 | 0.999 | 0.5 |
| Observed effect X 0.5 | 0.905 | 0.5 |
|  |  |  |
| Observed effect X 1 | 1 | 0.8 |
| Observed effect X 0.75 | 1 | 0.8 |
| Observed effect X 0.5 | 0.939 | 0.8 |

For the interaction effect of beta power and condition type:

**Supplementary table 2** Results from sensitivity power analysis for the interaction effect of beta power and condition type at a varying effect size multiplier.

| **Effect size multiplier** | **Power for Beta X Condition type** | **tau** |
| --- | --- | --- |
| Observed effect X 1 | 1 | 0.2 |
| Observed effect X 0.75 | 0.985 | 0.2 |
| Observed effect X 0.5 | 0.922 | 0.2 |
|  |  |  |
| Observed effect X 1 | 1 | 0.5 |
| Observed effect X 0.75 | 0.975 | 0.5 |
| Observed effect X 0.5 | 0.761 | 0.5 |

These results indicate that the study was well powered (≥ 90%) to detect effects even at half the magnitude of the observed estimates, demonstrating that the sample size was adequate for the primary analyses.

**Supplementary Figure 1**


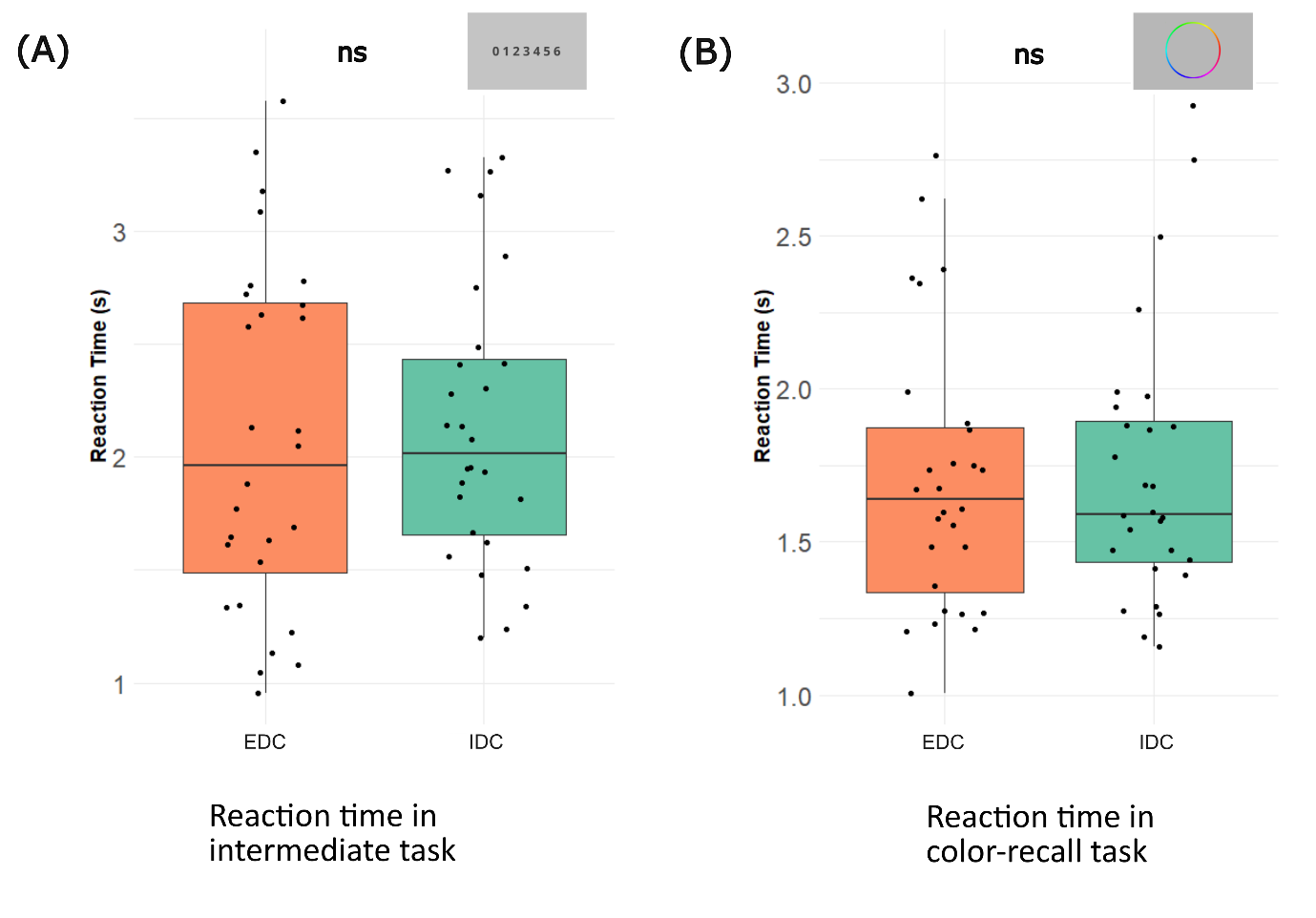


**Supplementary Figure 1** Reaction times in intermediate task and color-recall task. Personality adjectives were restricted to words containing two to four vowels in EDC condition, based on pilot testing to equalize reaction times across conditions and maintain a consistent overall delay duration. (A) Reaction time in intermediate task. (B) Reaction time in color-recall task.

**Supplementary Figure 2**


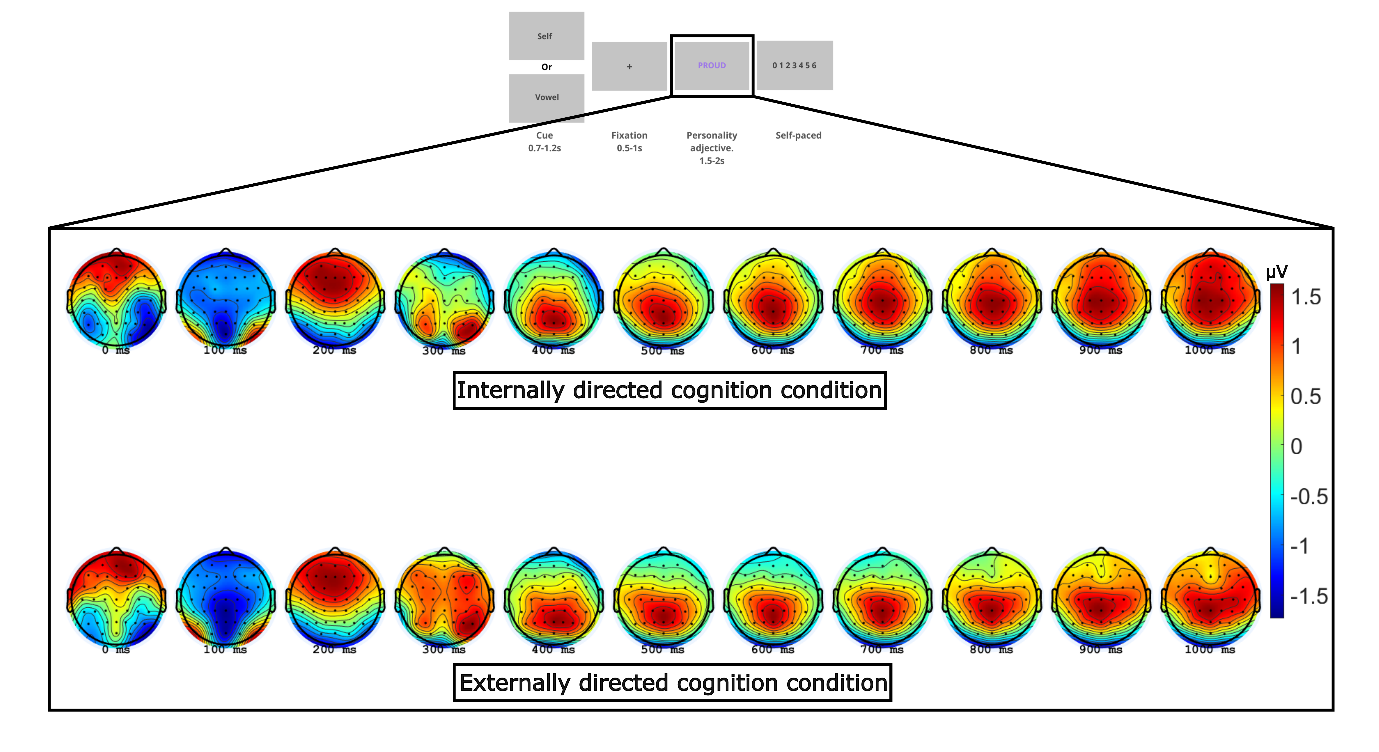


**Supplementary Figure 2** Scalp distribution of instantaneous voltage during the encoding epoch. The top row shows voltage distribution during self-referential processing of the personality adjective, while the bottom row shows voltage distribution during vowel-counting of the adjectives. Voltages are averaged over trials and across participants.

**Supplementary Figure 3**


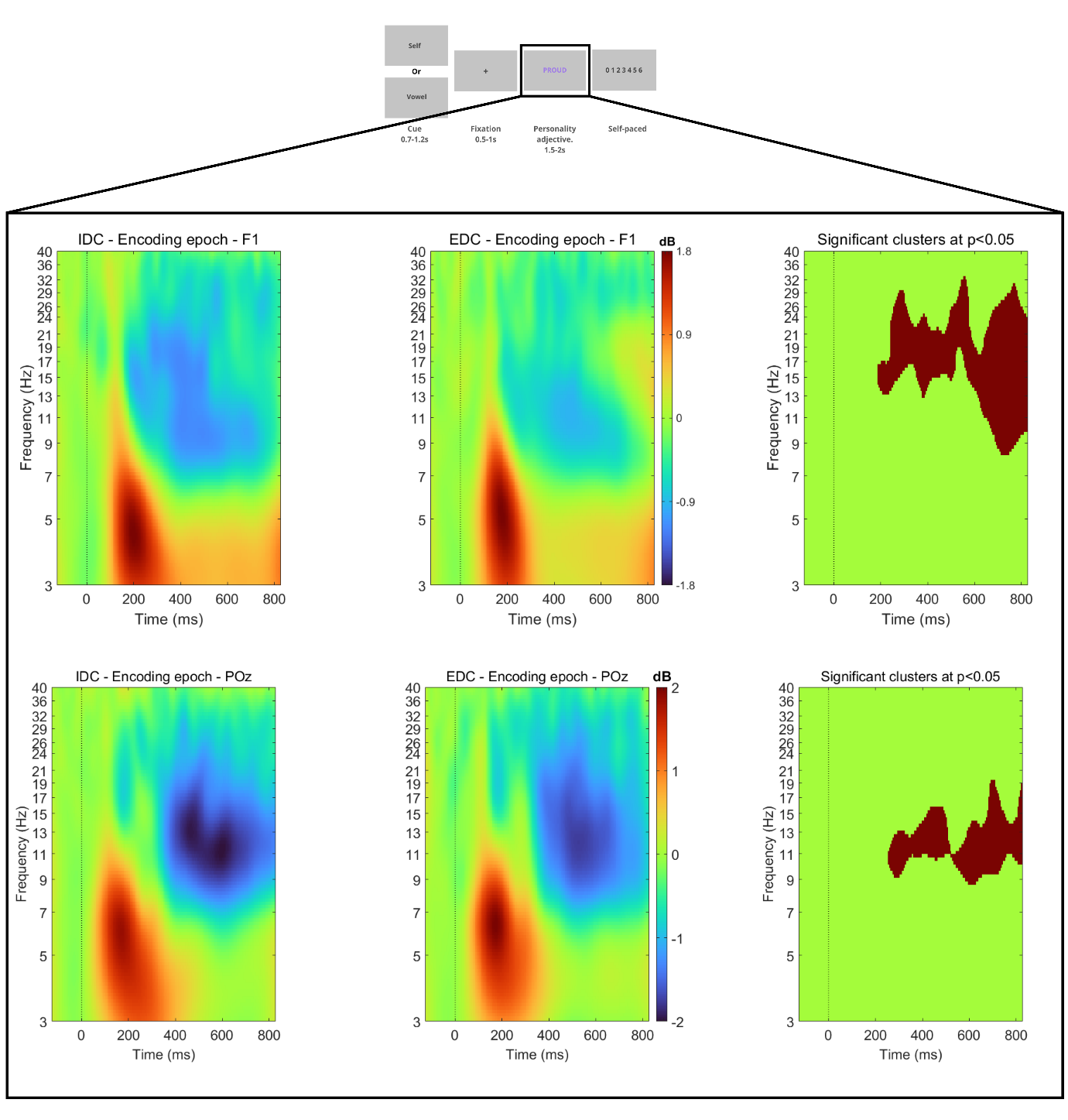


**Supplementary Figure 3** Time-frequency representations during the encoding epoch at frontal (F1; top row) and parietal (POz; bottom row) electrodes separately for each condition. Left and middle panels show power changes (in dB) for the Internally Directed Cognition (IDC) and Externally Directed Cognition (EDC) conditions, respectively. Right panels show significant time-frequency clusters (p < 0.05, cluster-corrected) comparing IDC and EDC. Red regions indicate statistically significant differences between conditions. Power values are baseline-corrected and averaged over trials and participants.

**Supplementary Table 3** List of personality adjectives sourced from (Anderson, 1968). Based on an earlier study (Davey et al., 2016), words centered around the median ‘likeableness’ rating reported in the original dataset were selected. The adjectives were matched on valence. In the EDC condition, adjectives were restricted to words containing two to four vowels, based on pilot testing to equalize reaction times across conditions and maintain a consistent overall delay duration (see Supplementary Figure 1).

| **Adjectives in IDC** |  | **Adjectives in EDC** |  |
| --- | --- | --- | --- |
| SILLY | DAYDREAMER | CRITICAL | UNHAPPY |
| SELFISH | CALM | UNHEALTHY | STUBBORN |
| OPPORTUNIST | UNPOPULAR | SARCASTIC | ALERT |
| IDEALISTIC | MEDIOCRE | CONFIDENT | PATIENT |
| EMOTIONAL | SUPERSTITIOUS | RESERVED | MODEST |
| OPTIMISTIC | ADVENTUROUS | RELAXED | INSECURE |
| CRAFTY | WORRYING | UNLUCKY | UNTIDY |
| SELF-CONSCIOUS | BOLD | DARING | LONELY |
| UNTRUTHFUL | PROGRESSIVE | DEPENDENT | OUTGOING |
| ANGRY | AGGREABLE | PROUD | CHOOSY |
| IMPOLITE | IMPULSIVE | ETHICAL | CURIOUS |
| CLEAR-HEADED | SELF-CRITICAL | QUIET | FEARLESS |
| ANNOYING | UNPREDICTABLE | PRACTICAL | QUICK |
| ABSENT-MINDED | PERSUASIVE | HOPEFUL | FORGETFUL |
| TALKATIVE | ENTERTAINING | AVERAGE | MORAL |
| NEAT | UNOBSERVANT | GREEDY | SYSTEMATIC |
| SHY | REALIST | BORING | STUDIOUS |
| PERFECTIONISTIC | CLUMSY | PERSISTENT | CONSISTENT |
| DISHONEST | SELF-CONFIDENT | SILENT | CHILDISH |
| CAUTIOUS | UNCONVENTIONAL | ANXIOUS | EASYGOING |
| UNFRIENDLY | POSSESSIVE | NERVOUS | MOODY |
| AGGRESSIVE | TIDY | TENSE | FEARFULL |
| INCONSISTENT | UNEMOTIONAL | TOUGH | TALENTED |
| NARROW-MINDED | SERIOUS | WORRIER | DIRECT |
| COURAGEOUS | HUMBLE | HESITANT | ABUSIVE |
| AGRUMENTATIVE | OVERSENSITIVE | NOISY | AIMLESS |
| REBELLIOUS | OBEDIENT | ORDERLY | EXCITED |
| UNPUNCTUAL | STRICT | CHARMING | CRUEL |
| UNIMAGINATIVE | LAZY | SKILLFUL | RESTLESS |
| MATERIALISTIC | SENTIMENTAL | UNFAIR | CAREFUL |

**Supplementary Table 4** Quantile regression results across three quantiles (τ = 0.2, 0.5, 0.8). In addition to the variables specified in Model 2 of the Methods section 2.7, this model included word length as a fixed covariate. Word length did not have any significant effect on color-recall performance at any quantile. The direction and magnitude of other significant effects remain unchanged to the original model.

| Tau | Coefficient | Estimate | Std. Error | Adjusted P value (FDR corrected) |
| --- | --- | --- | --- | --- |
| 0.2 | (Intercept) | 6.694507 | 1.484007 | 0.00045457 |
|  | LPP | 0.009745 | 0.031084 | 0.828907664 |
|  | Beta_encoding_ | 0.126465 | 0.074108 | 0.223214331 |
|  | AlphaF1_encoding_ | 0.021708 | 0.035509 | 0.679741616 |
|  | AlphaPOz_encoding_ | -0.05965 | 0.043898 | 0.360689802 |
|  | RT_I_ | 0.11246 | 0.19143 | 0.68057256 |
|  | RT_C_ | -0.71437 | 0.430257 | 0.232280883 |
|  | Word Length | -0.14526 | 0.077081 | 0.184058139 |
|  | **ConditionID** | **-6.15279** | **1.630068** | **0.002786545** |
|  | LPP: ConditionID | 0.03165 | 0.045227 | 0.653897699 |
|  | **Beta_encoding_: ConditionID** | **-0.25782** | **0.080325** | **0.010556676** |
|  | AlphaF1_encoding_: ConditionID | 0.003536 | 0.057217 | 0.950967845 |
|  | AlphaPOz_encoding_: ConditionID | -0.01534 | 0.07685 | 0.88184133 |
|  | RT_I_: ConditionID | 0.509745 | 0.282772 | 0.205390313 |
|  | **RT_C_: ConditionID** | **2.322206** | **0.664214** | **0.005697405** |
| 0.5 | (Intercept) | 6.631591 | 2.497469 | 0.036896475 |
|  | LPP | 0.022481 | 0.064442 | 0.819772541 |
|  | Beta_encoding_ | 0.086367 | 0.125346 | 0.653897699 |
|  | AlphaF1_encoding_ | 0.04673 | 0.090253 | 0.718759078 |
|  | AlphaPOz_encoding_ | -0.0832 | 0.068407 | 0.397577393 |
|  | RT_I_ | 0.736547 | 0.372262 | 0.160498808 |
|  | RT_C_ | 1.197147 | 1.029965 | 0.404403369 |
|  | Word Length | 0.124843 | 0.142321 | 0.558380699 |
|  | **ConditionID** | **-8.29031** | **1.666411** | **0.000189669** |
|  | LPP: ConditionID | -0.05399 | 0.072301 | 0.645172273 |
|  | **Beta_encoding_: ConditionID** | **-0.47095** | **0.164663** | **0.02327662** |
|  | AlphaF1_encoding_: ConditionID | -0.02277 | 0.130781 | 0.882121571 |
|  | AlphaPOz_encoding_: ConditionID | -0.05331 | 0.119617 | 0.758986742 |
|  | RT_I_: ConditionID | 0.442932 | 0.35825 | 0.397577393 |
|  | **RT_C_: ConditionID** | **4.224212** | **0.905784** | **0.000363925** |
| 0.8 | (Intercept) | 6.959484 | 2.150576 | 0.010556676 |
|  | LPP | -0.13056 | 0.082952 | 0.26131143 |
|  | Beta_encoding_ | -0.03491 | 0.174582 | 0.88184133 |
|  | AlphaF1_encoding_ | -0.11842 | 0.089592 | 0.360689802 |
|  | AlphaPOz_encoding_ | -0.16799 | 0.144805 | 0.404403369 |
|  | **RT_I_** | **1.970028** | **0.480886** | **0.001175614** |
|  | **RT_C_** | **5.45892** | **1.791759** | **0.015220719** |
|  | Word Length | 0.281013 | 0.208734 | 0.360689802 |
|  | **ConditionID** | **-9.17545** | **2.208038** | **0.001167345** |
|  | LPP: ConditionID | 0.305047 | 0.125057 | 0.059092262 |
|  | Beta_encoding_: ConditionID | -0.2414 | 0.260311 | 0.537443582 |
|  | AlphaF1_encoding_: ConditionID | 0.237834 | 0.137173 | 0.223092339 |
|  | AlphaPOz_encoding_: ConditionID | -0.19032 | 0.186225 | 0.48382039 |
|  | RT_I_: ConditionID | 0.404096 | 0.621499 | 0.666775567 |
|  | **RT_C_: ConditionID** | **6.348248** | **1.137113** | **4.60E-05** |

**References**

Brysbaert, M., & New, B. (2009). Moving beyond Kučera and Francis: A critical evaluation of current word frequency norms and the introduction of a new and improved word frequency measure for American English. *Behavior Research Methods, Instruments & Computers, 41*(4), 977–990. [https://doi.org/10.3758/BRM.41.4.977](https://psycnet.apa.org/doi/10.3758/BRM.41.4.977)

Brysbaert, M., Warriner, A.B. & Kuperman, V. Concreteness ratings for 40 thousand generally known English word lemmas. *Behav Res***46**, 904–911 (2014). <https://doi.org/10.3758/s13428-013-0403-5>

Kumle, L., Võ, M.LH. & Draschkow, D. Estimating power in (generalized) linear mixed models: An open introduction and tutorial in R. *Behav Res* **53**, 2528–2543 (2021). https://doi.org/10.3758/s13428-021-01546-0
